# Enzybiotics LYSSTAPH-S and LYSDERM-S as Potential Therapeutic Agents for Chronic MRSA Wound Infections

**DOI:** 10.3390/antibiotics9080519

**Published:** 2020-08-15

**Authors:** Lukáš Vacek, Šárka Kobzová, Richard Čmelík, Roman Pantůček, Lubomír Janda

**Affiliations:** 1 Clinical Immunology and Immunology of Infectious Diseases, Veterinary Research Institute, Brno, Hudcova 70, 62100 Brno, Czech Republic; vacek@vri.cz (L.V.); kobzova@vri.cz (Š.K.); 2 Department of Microbiology, St. Anne’s University Hospital Brno and Faculty of Medicine, Masaryk University, Brno, Pekařská 53, 65691 Brno, Czech Republic; 3 Institute of Analytical Chemistry of the Czech Academy of Sciences, Veveří 97, 60200 Brno, Czech Republic; cmelik@iach.cz; 4 Department of Experimental Biology, Faculty of Science, Masaryk University, Kamenice 5, 62500 Brno, Czech Republic; pantucek@sci.muni.cz

**Keywords:** enzyme therapy, enzybiotics, lysostaphin, LYSSTAPH-S, endolysin, LYSDERM-S, MRSA

## Abstract

Antibacterial antibiotic therapy has played an important role in the treatment of bacterial infections for almost a century. The increasing resistance of pathogenic bacteria to antibiotics leads to an attempt to use previously neglected antibacterial therapies. Here we provide information on the two recombinantly modified antistaphylococcal enzymes derived from lysostaphin (LYSSTAPH-S) and endolysin (LYSDERM-S) derived from kayvirus 812F1 whose target sites reside in the bacterial cell wall. LYSSTAPH-S showed a stable antimicrobial effect over 24-h testing, even in concentrations lower than 1 µg/mL across a wide variety of epidemiologically important sequence types (STs) of methicillin-resistant *Staphylococcus aureus* (MRSA), especially in the stationary phase of growth (status comparable to chronic infections). LYSDERM-S showed a less potent antimicrobial effect that lasted only a few hours at concentrations of 15 μg/mL and higher. Our data indicate that these antimicrobial enzymes could be of substantial help in the treatment of chronic MRSA wound infections.

## 1. Introduction

Antibiotic resistance poses an increased threat for patients’ health, prolongs stays in hospitals, and consumes more healthcare resources. In response to this threat, the WHO in 2015 announced the Global action plan on antimicrobial resistance, which sets out strategic objectives to reduce antimicrobial resistance impacts. According to this effort, research and development are needed to produce a novel approach to treatments that could be deployed against antibiotic-resistant infections [1,2,3,4,5,6]. 

Following this endeavor, this article focuses on promising antimicrobial enzymes LYSSTAPH-S (lysostaphin derivative) and LYSDERM-S (kayvirus 812F1 endolysin derivative) and their antibacterial potential against epidemiologically important sequence types (ST) of methicillin-resistant *Staphylococcus aureus* commonly found in chronic infections. 

**Figure 1:**
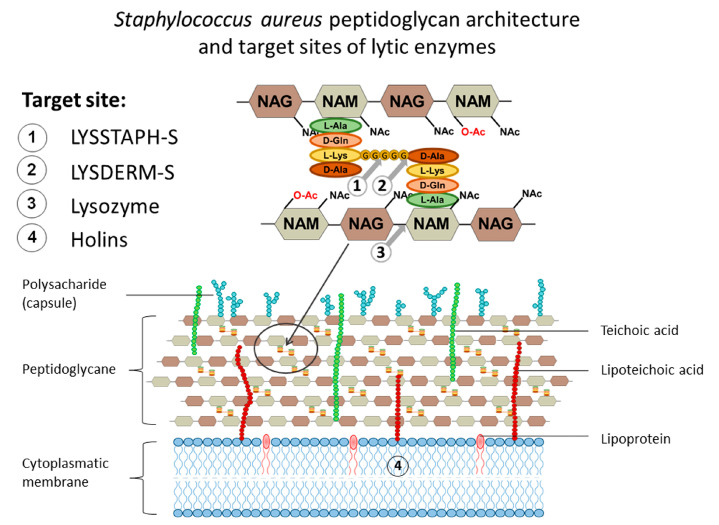
The cell wall of *Staphylococcus aureus* with the detailed structure of peptidoglycan. Cleavage sites of endolysin, lysostaphin, and lysozyme and target site of holins are marked 1 to 4 respectively.

Not surprisingly, the bacterial cell wall is the target of antibacterial therapies. The cell wall of *S. aureus* is primarily composed of peptidoglycan, teichoic acids, and various surface proteins. Peptidoglycan itself consists of glycan chains of alternating β-(1,4)-linked N-acetylglucosamine (NAG) and N-acetylmuramic acid (NAM; a variant of NAG with a D-lactate attached to the C-3 by an ether bond). Stem pentapeptides (L-Ala-D-iso-Gln-L-Lys-D-Ala-D-Ala) are covalently linked to the carboxyl carbon of the D-lactyl group of NAM, and interpeptide bridges (pentaglycines, i.e., chain of five glycine residues) connect the L-Lys component of one stem peptide to the D-Ala of a neighboring stem peptide, Figure 1. In this manner, glycan strands are cross-linked by stem peptides and pentaglycines and form a complex net of peptidoglycan (murein) sacculus [7]. 

Further, the antimicrobial enzymes lysostaphin and endolysin LysK, from which LYSSTAPH-S and LYDERM-S were derived, will be described.

### 1.1. Lysostaphin

Lysostaphin (EC 3.4.24.75), a glycyl-glycine endopeptidase, is a bacteriocin secreted by the Gram-positive bacterium *Staphylococcus simulans*. Bacteriocins, in general, are antibacterial proteins (and peptides) produced by bacteria as a competitive advantage over closely related species and cause growth inhibition or kill other bacterial species. Lysostaphin possesses bacteriolytic activity against staphylococci with pentaglycine interpeptide bridges because it specifically cleaves the bond between two glycines in the interpeptide bridge. As mentioned above, this is a distinct feature of the *S. aureus* cell wall and, thus, target selectivity is provided. Though its discovery and antimicrobial activity against *S. aureus* has been reported as early as the 1960s by Schindler and Schuhardt, its X-ray crystal structure has been reported only recently. Mature lysostaphin consists of an N-terminal catalytic domain with Zn^2+^-coordinating amino acid residues and a C-terminal cell wall targeting domain [8,9].

As shown earlier, every clinical application of the antimicrobial drug so far involved the development of antimicrobial resistance. Mechanisms associated with lysostaphin resistance usually involve mutations in *fem* genes. Fem factors catalyze the non-ribosomal synthesis of pentaglycine cross-bridges, which are essential for the addition of glycine residues 2 and 3 (FemA) and 4 and 5 (FemB). Mutations affecting *femA* renders this gene non-functional, resulting in the formation of monoglycine cross-bridges instead of pentaglycine ones, thus conveying partial or complete resistance to lysostaphin. Resistance can also be achieved by the incorporation of serines in place of some glycines in the cross-bridge [10,11,12]. 

Morikawa et al. [13] also identified the *sigB* gene as a regulator influencing the cell wall thickness. Cells depleted in *sigB* developed thinner envelopes and demonstrated increased sensitivity to lysostaphin. Conversely, overexpression of *sigB* led to increased resistance to lysostaphin [13]. In addition, Koehl et al. [14] connected an increased lysostaphin resistance of *S. aureus* to the thickened cell wall found in vancomycin intermediate-resistant *S. aureus* (VISA) strains, and also to a decreased autolytic activity. Gründling et al. [15] later identified another gene, *lyrA*, that caused a high degree of lysostaphin resistance [13,14].

Even though there are several reports of resistance development in *S. aureus* to lysostaphin, it seems that every resistance mechanism has its significant drawbacks. Kusuma et al. [11] demonstrated that mutations in *femA* led to reduced growth rate and virulence in a mouse kidney infection model in comparison to their wild-type parental strains. Moreover, other work showed a clear relationship between *femA*-mediated lysostaphin resistance and increased susceptibility to β-lactams. It has been shown that the modified transpeptidase–penicillin-binding protein 2a (PBP 2a) encoded by the *mecA* gene, which is responsible for β-lactam resistance in MRSA strains, cannot perform its function when only monoglycine cross-bridges are present. However, the regular PBP 2 transpeptidase is still capable of crosslinking stem peptides by monoglycine bridges. However, as a result of the situation, conventional beta-lactam antibiotics may inhibit PBP 2, so that the resulting strains are again susceptible to methicillin [16,17,18]. 

### 1.2. Endolysin LysK

The LysK endopeptidase is one of the characterized endolysins derived from phage K, a large broad-spectrum staphylococcal myovirus from the genus *Kayvirus*, recently reclassified to family of *Herelleviridae*. It consists of three domains typical for staphylococcal phage endolysins, the Cysteine, Histidine-dependent Amidohydrolase/Peptidase (CHAP) domain, the Amidase-2 domain (Ami-2), and the Src homology 3 - prokaryotic homologue (SH3b) domain. The cleavage site of the CHAP domain (d-Ala–Gly) and amidase domain (MurNAc–l-Ala) were revealed by mass spectrometry (MS) and the analysis of peptidoglycan digestion products. The SH3b cell wall binding domain is known to bind to the pentaglycine bridge, and similar activity was shown for the SH3b-like cell wall targeting domain of the lysostaphin. Further, the deletion construct consisting of the CHAP and SH3b domains, without the amidase domain was demonstrated to possess similar antibacterial activity as the full-length enzyme [19,20]. 

LysK (in some articles, also referred to as GH15, Sb-1, G1, SA11, A5W, or 812) is one of the most potent staphylococcal phage endolysins with a broad-spectrum antibacterial activity. It has the capability to kill various strains of *S. aureus*, including MRSA and VRSA strains, as well as coagulase-negative staphylococci. Interestingly, the CHAP domain was also shown to have an even broader lytic spectrum than original LysK, with the activity towards bacteria of genera *Streptococcus*, *Micrococcus*, *Arthrobacter,* and others [19,21]. 

In has been hypothesized that during the co-evolution of phages and their bacterial hosts, endolysins have developed their activity toward conserved cell wall structures essential for survival, which makes the antibacterial resistance a rare event. As described earlier, the majority of resistance mechanisms are focused within the cell, and the extracellular application of endolysins hampers the possibility to develop resistance. Indeed, to our knowledge, bacterial resistance to endolysins has not yet been described. Nevertheless, researchers recently attempted to further reduce the possibility of resistance development by using fusion enzymes consisting of catalytic domains of LysK and lysostaphin [22,23,24]. 

## 2. Results

### 2.1. Enzyme Preparation

In this work, we used a new recombinant variant of lysostaphin - LYSSTAPH-S, which is characterized by a gene sequence optimized for heterologous expression in *E. coli*. The original amino acid sequence (WP_013012297.1) was truncated by 209 amino acids (predomain). The less hydrophobic methionine 237 was replaced by isoleucine [25]. The resulting recombinant DNA construct contained a ubiquitin gene, a 14× histidine anchor, a catalytic domain gene, and a cell wall binding domain gene. The entire fusion protein contains 356 amino acids with a total molecular weight of 39.163 kDa. 

Further, we used a novel recombinant variant of lysostaphin LYSDERM-S optimized for heterologous expression in *E. coli*. The amino acid sequence for endolysin is based on the LysF1 construct [26]. The final construct contains a ubiquitin gene, a 14× histidine anchor, a CHAP domain, and a cell wall binding gene (SH3b domain). The entire fusion protein contains 395 amino acids with a total molecular weight of 43.653 kDa.

The protein production was comparable to other expression systems and achieved yields of 200 mg/L in both tested enzymes. The SDS-PAGE electrophoresis results of recombinant proteins LYSDERM-S and LYSSTAPH-S are depicted in Supplementary Material Figure S1. 

### 2.2. Plate Lysis Assay

**Figure 2:**
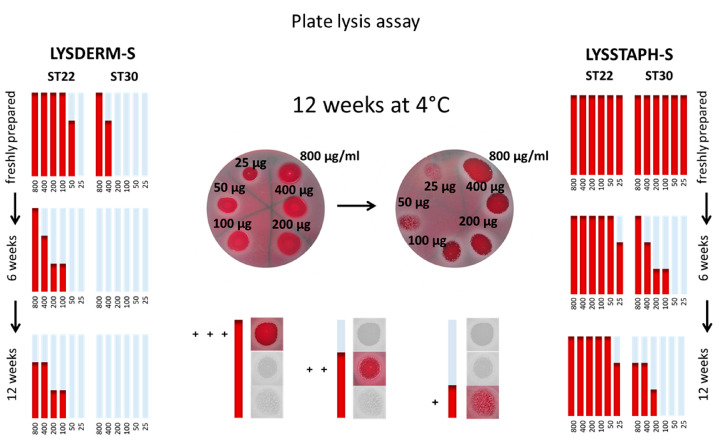
Antibacterial susceptibility of *Staphylococcus aureus* sequence types (ST) ST22 and ST30, at three different time points, freshly prepared, after six weeks of storage at 4 °C, and after 12 weeks of storage at 4 °C. LYSSTAPH-S and LYSDERM-S concentrations used: 800, 400, 200, 100, 50, 25 μg/mL. Clear zones were assessed as “+++”, zones with minimal bacterial growth as “++”, zones with substantial bacterial growth as “+”, and no visible zone as “-”.

The antibacterial effect on selected *S. aureus* strains was evaluated semiquantitatively, as depicted in Figure 2. Clear zones were assessed as “+++”, zones with minimal bacterial growth as “++”, zones with substantial bacterial growth as “+”, and no visible zone as “-“. The results were transformed into graphs for a simple comparison between strains and times of storage. Figure 2 shows results for *S. aureus* sequence types ST22 and ST30 at three different time points, freshly prepared, after six weeks of storage at 4 °C, and after 12 weeks of enzyme solution storage at 4 °C. The full results of the other tested *S. aureus* strains are shown in Supplementary Material Figures S2 and S3. 

The results show a decrease in antibacterial susceptibility with prolonged time of storage, during which slow enzyme denaturation takes place. When the highest concentration of LYSSTAPH-S was used, the tested strains showed slightly reduced antimicrobial activity after 12 weeks (93.6%) of storage at 4 °C compared to freshly prepared solutions (100%) and after six weeks (100%). In contrast, LYSDERM-S showed a decrease in antibacterial susceptibility of the tested strains after six weeks (33.3%) compared to the freshly prepared enzyme (100%). After 12 weeks of storage, no strain was susceptible to the enzyme. Thus, the degradation of LYSDERM-S in time is more rapid in comparison with LYSSTAPH-S. The detailed results are shown in Supplementary Material Figures S2 and S3.

### 2.3. Growth Kinetic Analysis and Cluster Analysis

The cultivation of *S. aureus* strains without LYSSTAPH-S or LYSDERM-S presence did not show any significant difference in the growth rate among the tested strains, and all of the strains reached stationary phase after 4 h of cultivation.

**Figure 3:**
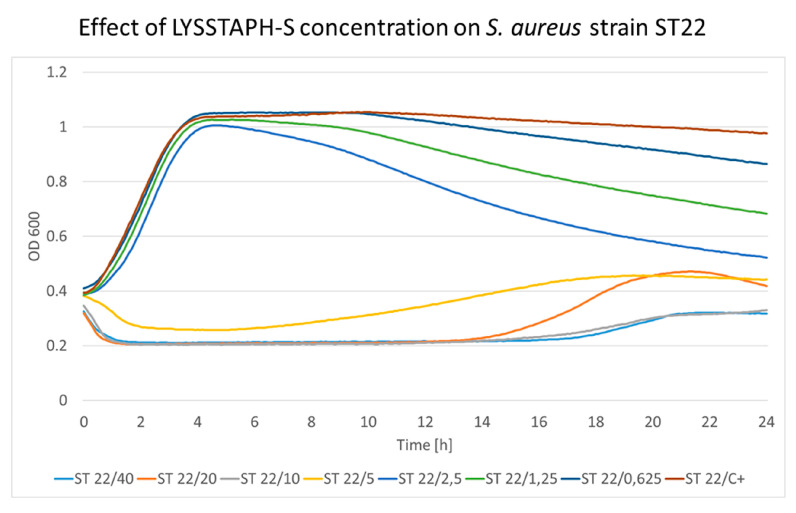
The concentration effect of the added LYSSTAPH-S on *S. aureus* ST22 strain. Concentrations ranging from 0.625 to 2.5 μg/mL affect the growth curve only when the stationary phase is reached. The decrease in optical density is concentration-dependent (particular concentration in μg/mL is mentioned after the slash sign). ST (sequence type of *S. aureus*), C+ (growth control).

**Figure 4:**
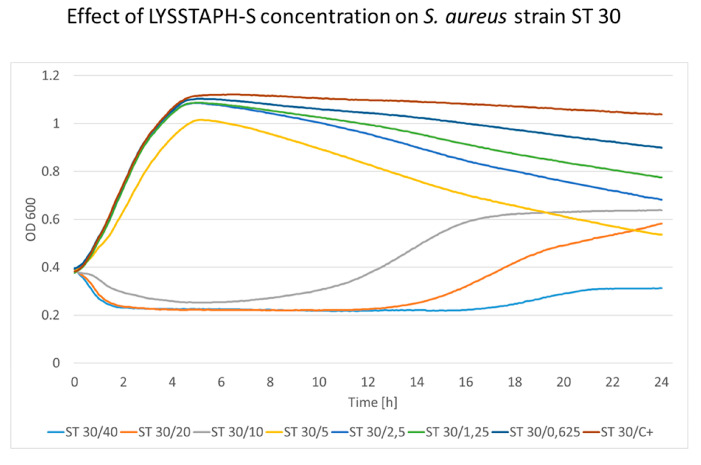
The concentration effect of added LYSSTAPH-S on *S. aureus* ST30 strain. Concentrations ranging from 0.625 to 5 μg/mL affect the growth curve only when the stationary phase is reached. The decrease in optical density is concentration-dependent (particular concentration in μg/mL is mentioned after the slash sign). ST (sequence type of *S. aureus*), C+ (growth control).

The cultivation of *S. aureus* strains with increasing LYSSTAPH-S concentrations showed a distinct pattern present in all strains. Bacterial growth was affected by LYSSTAPH-S concentrations ranging from 0.625 to 2.5 μg/mL only when the stationary phase was reached. This is probably due to the rapid growth at the beginning of the experiment when nutrition was abundantly present in the growth medium. When the stationary phase was reached and cellular growth and division were limited, the lytic effect of the LYSSTAPH-S outweighed the peptidoglycan construction and the repair mechanisms and bacterial cells were disrupted, thus the optical density of the medium decreased. This effect is depicted in Figure 3 and Figure 4 for ST22 and ST30, respectively. Higher concentrations partially or entirely inhibited bacterial growth. The full results of the other tested *S. aureus* strains are shown in Supplementary Material Figure S4. 

The background optical density (OD) for the sterile cultivation medium was 0.207 ± 0.021 in our experiments. After the initial decrease of OD, which could be seen in earlier time points when high concentrations of the enzyme were used, OD levels dropped to the OD level of the background (sterile cultivation medium). Therefore, the formation of the degradation products and the possible interference with the OD measurement remained below the detection limit of this method. This implies that the degradation products had minimal to no effect on the OD measurement during this experiment. 

Elevated levels of the OD at later time points suggested partially decreased activity of LYSSTAPH-S, which allowed for bacterial growth because nutrients in the cultivation medium had not been depleted yet. When nutrients were eventually depleted, and bacteria could not promptly grow and repair damages to their cell wall, the OD values started to decrease again, since LYSSTAPH-S still maintained part of its original activity. This effect can be seen in Figure 3, the growth curve of ST22 in the presence of 20 μg/mL of LYSSTAPH-S (orange line).

**Figure 5:**
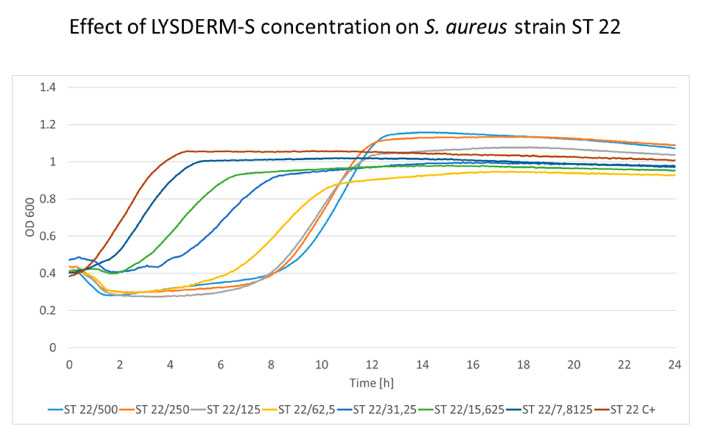
The concentration effect of added LYSDERM-S on *S. aureus* ST22 strain. Concentrations range from 7.8125 to 500 μg/mL; the decrease in optical density is concentration-dependent (particular concentration in μg/mL is mentioned after the slash sign). ST (sequence type of *S. aureus*), C+ (growth control).

**Figure 6:**
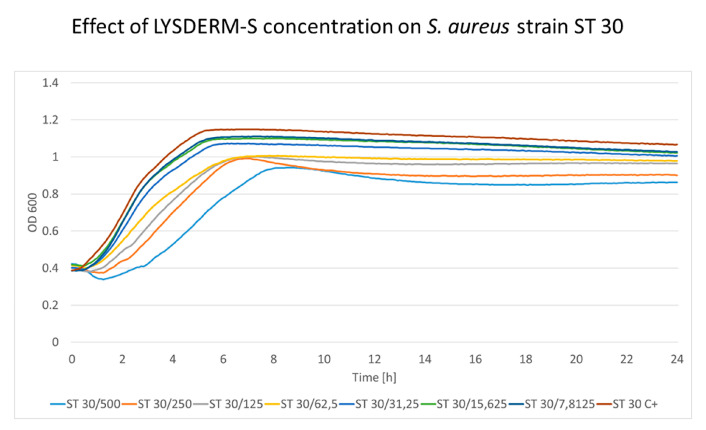
The concentration effect of added LYSDERM-S on *S. aureus* ST30 strain. Concentrations range from 7.8125 to 500 μg/mL; the decrease in optical density is concentration-dependent (particular concentration in μg/mL is mentioned after the slash sign). ST (sequence type of *S. aureus*), C+ (growth control).

The cultivation of *S. aureus* strains with increasing LYSDERM-S concentrations showed a concentration-dependent lytic effect. The bacterial growth was affected by LYSDERM-S concentrations ranging from 7.8125 to 500 μg/mL only at the beginning of the experiment. The inoculum effect could be seen in the later phase of the experiment (antimicrobial effect dependent on the number of bacteria initially inoculated into the assay, in this assay 2 × 10^8^ CFU/mL). Strains ST22 and ST30 are shown in Figure 5 and Figure 6, respectively. The full results of the other tested *S. aureus* strains are shown in Supplementary Material Figure S5. Strain ST30 (Figure 6) was not inhibited, even with the highest concentrations of LYSDERM-S tested (500 μg/mL). Other strains showed growth inhibition in the range of 15.625 to 62.5 μg/mL of LYSDERM-S concentration. 

Based on growth characteristics over 24 h, bacterial strains were subjected to cluster analysis and divided into five distinct groups, according to increasing resistance towards LYSSTAPH-S. *S. aureus* strains ST22, ST154, and ST225 were assigned to group 1 (increased susceptibility towards LYSSTAPH-S), ST8 and ST398 to group 2, ST7, ST15, ST45, ST80, and ST121 to group 3, two ST395 strains to group 4, and finally ST30 to group 5 (increased resistance towards LYSSTAPH-S). 

### 2.4. Bacterial Cluster Formation

*S. aureus* strains ST22, ST30, and ST395 belonging to different groups due to their growth inhibition by LYSSTAPH-S were inspected for bacterial cluster formation by two different methods, by microscopic examination and by spectrophotometric quantification of the Helm’s flocculation test. No significant difference (*p* = 0.198) among the tested strains was discovered (data not shown). 

### 2.5. Biofilm Formation Assay

**Figure 7:**
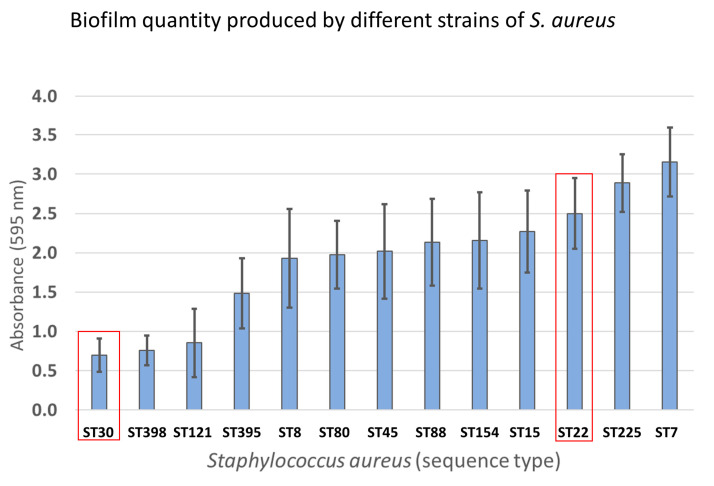
Quantification of produced biofilm of strains examined by growth curve analysis.

Quantification of produced biofilm in all strains examined by growth curve analysis was performed by the crystal violet biofilm assay. All tested strains showed medium to high biofilm formation. It is worth noting that strains determined as the most resistant toward LYSSTAPH-S and LYSDERM-S showed less tendency to form biofilms than the most susceptible strains (Figure 7). 

### 2.6. Resistance to Vancomycin

Reduced susceptibility to LYSSTAPH-S or LYSDERM-S due to increased vancomycin resistance was examined by minimal inhibitory concentration (MIC) determination. None of the tested strains were identified as vancomycin intermediate-resistant or vancomycin-resistant *S. aureus*. 

### 2.7. Peptidoglycan Analysis

**Figure 8:**
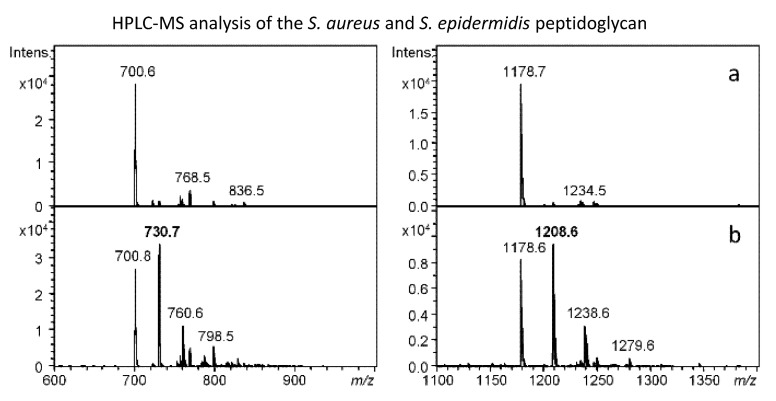
Mass spectrometry (MS) spectra of the peaks corresponding to selected peptides (left) and muropetides (right) derived from *Staphylococcus aureus* ST154 (**a**) and *Staphylococcus epidermidis* CCM 4418 (**b**) peptidoglycan after the action of LYSDERM-S. *S epidermidis*-specific ions are labeled with bold numbers corresponding to differences in the interpeptide bridge (30 Da represents substitution of glycine with serine residue).

Four samples of peptidoglycan of *S. aureus* strains ST15, ST22, ST30, and ST154, and one sample of *Staphylococcus epidermidis* CCM 4418 (=ATCC 12228) peptidoglycan were analyzed after the treatment with the lytic enzymes LYSSTAPH-S and LYSDERM-S. High-performance liquid chromatography-mass spectrometry (HPLC-MS) analyses of digested peptidoglycans did not find significant differences among *S. aureus* samples based on (muro) peptide composition. The reference *S. epidermidis* strain showed a significant difference (additional peaks “+30 Da”) from *S. aureus* peptidoglycan due to the increased number of serine residues in the interpeptide bridge composition of its peptidoglycan. Figure 8 represents the differences in the peptidoglycan composition as determined by HPLC-MS analysis.

## 3. Discussion

Novel antimicrobial agents are urgently needed for the reduction of antimicrobial resistance impacts. In this study, we present recombinant antimicrobial enzymes LYSSTAPH-S and LYSDERM-S and demonstrate their antimicrobial efficiency against the set of MRSA strains and their storage stability at 4 °C for enzyme solution. We optimized the production of LYSSTAPH-S and LYSDERM-S expressed in *E. coli* in the pUbEx15 vector to minimize the production time and costs. The protein production was comparable to other expression systems and achieved yields of 200 mg/L. Our production in a small-scale laboratory expression system exceeded conventional laboratory expression systems (20 mg/L) and reached concentrations commonly achieved at the semi-industrial level (300 mg/L) [27,28,29]. Our final goal was to achieve a yield close to the 5 g/L medium. We are currently testing an expression system capable of producing both recombinant proteins in a bioreactor with the future prospect of using these recombinantly prepared enzymes for topical application in chronic wound treatment. 

Chronic infections are generally less understood, but studies suggest that stationary-phase liquid bacterial cultures and chronic-wound fitness of bacteria correlate significantly. Morgan et al. [30] demonstrated this situation in the most predominant bacterial species found in chronic wounds, *Pseudomonas aeruginosa*. Therefore, we based our observations not only on the commonly used turbidity reduction assay, which monitors turbidity levels during the period of one to several hours [31] but prolonged this time up to 24 h. To our knowledge, there is no study that would closely monitor growth kinetics of MRSA strains in the presence of lysostaphin or endolysin LysK or their derivatives over a prolonged period of time in the high-throughput set-up of 384-well microplates. 

The growth kinetic analysis implies that LYSSTAPH-S is not affected by *S. aureus* defense mechanisms and antimicrobial activity is stably maintained over a 24-h long measurement. The growth kinetic analysis revealed that even concentrations lower than 1 µg/mL of LYSSTAPH-S significantly affect staphylococci in the stationary phase. This is particularly important for future applications in chronic infections management. Moreover, concentrations over 10 µg/mL are capable of lysing staphylococcal cells in the exponential phase of growth. These results indicate that higher concentrations of enzyme should be used to treat acute infections. 

Further, Wu et al. [32] recently pointed out the difference between the susceptibility of lysostaphin in the non-growth environment of phosphate-buffered saline (PBS) and the growth-supporting medium tryptic soy broth (TSB). They confirmed the low cleavage of pentaglycine bridges and low bacterial cell killing in TSB caused by lysostaphin. Our findings are in agreement with this result and we would like to note that bacteria in chronic wound infections are found in the state of high-density bacterial growth conditions and are supplemented by the limited supply of nutrients provided by wound exudate, and, therefore, reside in conditions closely resembling the stationary phase in the growth-supporting medium [30]. Considering this, LYSSTAPH-S seems to be a suitable candidate for further testing and, possibly, clinical trials in the field of chronic wound management. 

The growth kinetic analysis of LYSDERM-S suggests an initial antibacterial effect, as seen in LYSSTAPH-S, but this response is concentration-dependent, diminishes over time, and the number of bacteria rises again. The LYSDERM-S probably loses its activity within several hours of testing. Also, LYSDERM-S seems to be less effective towards some sequence types of *S. aureus*, namely ST30. Even the highest concentrations of LYSDERM-S could not reduce the initial numbers of this bacterial strain. 

The short-term antibacterial effect of LYSDERM-S could be explained by the inoculum effect (the inoculum density was higher than for a standard antibiotic susceptibility testing) and the enzyme stability. The initial high bacterial concentration might influence the later stages of kinetic analysis where bacterial counts rise again. This effect is well known in standard antimicrobial susceptibility testing [33]. Further, our previous assessment of the stability of the tested proteins by the melting curve analysis showed a difference of 20 °C in T_m_ between these two enzymes (data not shown). This would indicate that LYSDERM-S has a greater tendency to denature during growth kinetic analysis compared to LYSSTAPH-S. These hypotheses are also supported by the plate lysis assay repeated over a period of 12 weeks. For example, the previous studies showed that the addition of Ca^2+^ cations resulted in an enhanced (more than 100 times) stability of LysK endolysin [34], and we argue that further structural stabilization of LYSDERM-S by recombination might increase its antibacterial effectivity. We see the improvement of structural instability as an important goal for future research.

Considering other aspects, we found that the level of bacterial susceptibility was independent of other growth parameters tested, such as cluster formation or biofilm development. Also, the target site, pentaglycine bridge within the peptidoglycan, of these enzymes also seemed to be similar among the tested strains and did not show any variability across the tested strains. Therefore, it remains unclear where the basis of the different susceptibility lies.

Nevertheless, LYSSTAPH-S and LYSDERM-S antimicrobial enzymes proved to be efficient in killing of the diverse strains of methicillin-resistant *S. aureus*. As a result, we believe that even well studied antimicrobial enzymes, such as lysostaphin and endolysin LysK and their derivatives could be further modified in order to improve their antimicrobial properties and stability and ultimately serve as a worthy alternative to well-established antimicrobial therapy of methicillin-resistant *S. aureus* infections.

## 4. Materials and Methods 

### 4.1. Bacterial Strain Selection

*Staphylococcus aureus* strains were selected from the Collection of Microorganisms of the Department of Experimental Biology, Faculty of Science, Masaryk University, Czech Republic. Based on multilocus sequence typing (MLST) of human and veterinary *S. aureus* strains in this collection, candidate sequence type (ST) strains were selected to obtain the most heterogeneous representatives of each clonal cluster (CC). The selected representatives of *S. aureus* strains belonged to ST1, ST5, ST7, ST8, ST15, ST22, ST30, ST45, ST80, ST88, ST121, ST154, ST225, ST239, ST395, and ST398.

### 4.2. Enzyme Preparation

The expression vector pUbEx15 was constructed from the vector pET28 by insertion of special DNA cassettes by XbaI and BamHI restriction enzymes. The DNA cassette contains the optimized gene for heterologous expression in *E. coli* for ubiquitin, 14-His-Tag, and the target site for Tobacco Etch Virus (TEV) protease. The full sequences of the LYSSTAPH-S and LYSDERM-S genes were synthesized by GenScript Biotech (Leiden, The Netherlands), optimized for heterologous expression in *E. coli*, and inserted into the vector pUbEx15 by NcoI and NotI restriction enzyme [35]. 

Both enzymes LYSSTAPH-S and LYSDERM-S were produced in the *E. coli* strain BL21 (DE3) carrying pUbEx15 plasmid and grown in 1 L flasks containing 200 mL LB medium supplemented with 50 μg/mL kanamycin at 37 °C and 180 rpm. When the optical density (OD 600) reached 0.6, the recombinant protein expression was induced by the addition of IPTG to the final concentration of 0.4 mM, and the culture was further incubated for 16 h at 20 °C. The cells were harvested by centrifugation at 6000× *g* and 4 °C for 10 min., then resuspended in lysis buffer (100 mM Tris-HCl, pH = 8, with 400 mM NaCl, 0.1% Triton X-100 and lysozyme to the final concentration 10 μg/mL), and sonicated for 15 min on ice (1 s pulses with 4 s intervals, amplitude 35%; SonoPlus Badelin, (Berlin, Germany)). Both suspensions were centrifuged for 30 min at 15,000× *g* and 4 °C, filtered through 0.22 μm filter (Techno Plastic Products AG, Trasadingen, Switzerland), and the supernatants were used for protein purification. Both supernatants were loaded onto HisTrap equilibrated with 100 mM Tris-HCl buffer, pH = 8, with 400 mM NaCl, 10 mM imidazole. LYSSTAPH-S and LYSDERM-S were eluted with the same elution buffer (100 mM Tris-HCl, 400 mM NaCl and 300 mM imidazole). After purification, the proteins were dialyzed against an ammonium acetate buffer (0.1 M, pH = 5.5; Oxoid, UK). The protein concentrations were determined using the BCA microplate assay with bovine serum albumin (BSA) as a standard according to the manufacturer’s instructions (measured with Pierce™ BCA Protein Assay Kit, Thermo Scientific, Waltham, MA, USA). The presence of the target protein was further verified with preparative electrophoresis in a polyacrylamide gel (Model 491 Prep Cell, BioRad, Hercules, CA, USA) according to the manufacturer’s protocol. For protein expression and purity verification, the SDS-PAGE was used. The yield of both pure proteins calculated on 1 L of the growth medium was about 200 mg.

### 4.3. Plate Lysis Assay

Purified LYSSTAPH-S and LYSDERM-S solutions were serially diluted in distilled water to obtain final concentrations of 800, 400, 200, 100, 50, and 25 μg/mL. Each staphylococcal strain was cultivated overnight in 20 mL of meat peptone broth (MPB) prepared from nutrient broth 13 g/L, yeast extract 3 g/L, bacteriological pepton 5 g/L, pH = 7.4 (Oxoid, UK) at 37 °C with shaking. Overnight cultures were then diluted 1:500 in a fresh MPB and further cultivated for 4 h to reach the mid-exponential phase. The cells were harvested by centrifugation at 6000× *g* and 4 °C for 10 min. The cells were washed and resuspended in PBS buffer (Oxoid, UK) three times. Next, bacterial cultures were resuspended in PBS to obtain the final optical density of 0.5 McFarland standard and swabbed uniformly across Trypticase Soy Agar with 5% Sheep Blood culture plates (bioMérieux, Craponne, France). 10 μL of each purified LYSSTAPH-S dilution was spotted onto a freshly plated lawn of *S. aureus*. The spotted plates were incubated overnight at 37 °C. After 20–24 h of cultivation, inhibition zones were observed and semiquantitatively evaluated. LYSSTAPH-S and LYSDERM-S solutions were then stored at 4 °C for 6 and 12 weeks as enzyme solutions and experiments were repeated to re-evaluate stability and antibacterial effect. 

### 4.4. Growth Kinetic Analysis and Cluster Analysis

Purified LYSSTAPH-S was serially diluted in PBS to obtain stock concentrations of 400, 200, 100, 50, 25, 12.5, and 6.25 μg/mL. Purified LYSDERM-S was serially diluted in PBS to obtain stock concentrations of 5000, 2500, 1250, 625, 312.5, 156, 25, and 78.125 μg/mL. Each staphylococcal strain was cultivated overnight in 20 mL of Trypticase Soy Broth (TSB, Oxoid, UK) at 37 °C with shaking. Overnight cultures were then diluted 1:500 in a fresh TSB and further cultivated for 4 h to reach the mid-exponential phase. Bacterial cultures were then diluted with fresh TSB to obtain a bacterial suspension of the final optical density of (OD_600_) = 0.2 (approx. 2 × 10^8^ CFU/mL). 90 μL of each strain suspension and 10 μL of each LYSSTAPH-S and LYSDERM-S stock solutions were mixed in Nunc^®^ 384-well microplates (Sigma-Aldrich, St. Louis, MO, USA) in triplicates and sealed with sterile ThermalSeal^®^ films (Sigma-Aldrich, St. Louis, MO, USA). 10 μL PBS was added to each strain suspension to serve as growth control. Microplates were incubated at 37 °C with shaking in Tecan Infinite M200 PRO microplate reader (Tecan Trading AG, Trasadingen, Switzerland), and optical density was measured (A = 600 nm) every 5 min for 24 h. The cultivation was repeated three times to obtain nine growth curves for each bacterial strain and LYSSTAPH-S or LYSDEERM-S dilution. 

Average growth curves were obtained for each bacterial strain and lysostaphin dilution and compared among themselves. Dendrogram of average growth curves was obtained to help to assess the ideal number of clusters for cluster analysis (TIBCO Statistica 13.5, TIBCO Software Inc., New York, NY, USA). Next, bacterial strains were divided into five groups based on the obtained dendrogram and empirical evaluation (cluster analysis algorithm: k-Means, number of iterations: 50, distance method: Euclidean distances; TIBCO Statistica 13.5, TIBCO Software Inc., USA), and representative members of groups with the most dissimilar growth curve timelines were selected for further analyses. 

### 4.5. Bacterial Cluster Formation

Bacterial cluster formation was inspected in *S. aureus* strains ST22, ST30, and ST395 by two different methods. The first method relied on the microscopical evaluation of clusters. In brief, 20 μL of each overnight bacterial suspension (cultivation in TSB at 37 °C) was directly stained by adding 5 μL of Alcian Blue 8 GX (Sigma-Aldrich, St. Louis, MO, USA) to avoid possible cluster disruption. Specimens were visually examined by a 40× phase-contrast objective (magnification 400×, Nikon ECLIPSE Ci-E, Nikon Corp., Tokyo, Japan) to assess the number and size of clusters. 

In the second method, the methodology used for the spectrophotometric quantification of Helm’s flocculation test [36] was adopted. In brief, bacterial suspensions were obtained as described in the Plate Lysis Assay, with the bacterial cultivation in TSB instead of MPB. Optical density (OD 600) of 2 mL of each bacterial suspension was measured immediately in disposable cuvettes. Further, bacterial suspensions in the disposable cuvettes were let to settle freely for 3 h at ambient temperature. After the given time, 500 μL of the upper portion of the suspensions were taken, optical density at 600 nm was measured again and compared to the OD value before the settling period. The experiment was repeated three times in triplicates. The differences in OD values for bacterial strains were compared by one-way ANOVA (TIBCO Statistica 13.5, TIBCO Software Inc., New York, NY, USA). 

### 4.6. Biofilm Formation Assay

The biofilm formation assay followed the standard methodology described in Stepanović et al. [37]. In brief, bacterial strains were grown overnight in TSB and then diluted 1:500 in a fresh TSB and further cultivated for 4 h to reach the mid-exponential phase. Bacterial cultures were then diluted with fresh TSB to obtain bacterial suspensions of optical density 0.5 McFarland standard (approx. 1–2 × 10^8^ CFU/mL). Then bacterial suspensions were further diluted 1:100 with TSB medium supplemented with 1% glucose. A total of 200 μL of each bacterial suspension was pipetted into microtiter plates in triplicates and incubated for 20 h at 37 °C. After the incubation, the bacterial suspension was carefully removed, and the biofilm was gently washed three times with PBS. The biofilm was fixed by heat at 37 °C overnight and subsequently stained with a 1% crystal violet solution (Sigma-Aldrich, St. Louis, MO, USA) for 20 min. The excess of the staining solution was removed by rinsing in running water. Bound crystal violet was resuspended in 200 μL of 33% acetic acid (Sigma-Aldrich, St. Louis, MO, USA), and the absorbance at 595 nm was determined using a Tecan Infinite M200 PRO microplate reader (Tecan Trading AG, Trasadingen, Switzerland). 

### 4.7. Resistance to Vancomycin

As described earlier, vancomycin-resistant strains exhibit reduced susceptibility to lysostaphin. To examine this effect, minimal inhibitory concentrations (MIC) were determined for each *S. aureus* strain following the broth microdilution methodology according to European Committee on Antimicrobial Susceptibility Testing (EUCAST) recommendations. In brief, bacterial cell suspensions of all selected strains were prepared (5 × 10^5^ CFU/mL) in Mueller–Hinton broth (Oxoid, UK) and commercial MIC sets containing vancomycin in concentrations ranging from 0.5 to 32 mg/L in two-fold dilutions were inoculated. MIC sets were cultivated for 20 h at 37 °C and subsequently visually inspected for turbid growth. MIC values were determined as the first well with no visible turbid growth. VISA strains were characterized by MIC in the range of 4 to 8 mg/L, vancomycin-resistant *S. aureus* (VRSA) strains were characterized by MIC > 8 mg/L. 

### 4.8. Preparation of Bacterial Peptidoglycan

*Staphylococcus aureus* strains ST15, ST22, ST30, and ST154 were used for peptidoglycan analysis and *Staphylococcus epidermidis* CCM 4418 (=ATCC 12228) served as a reference control strain. Staphylococcal strains were cultivated in 200 mL of meat peptone broth (MPB) prepared from nutrient broth 13 g/L, yeast extract 3 g/L, bacteriological peptone 5 g/L, pH = 7.4 (Oxoid, UK) at 37 °C with shaking to a final OD_600_ = 1.5. Bacteria were centrifuged at 5000× *g* and resuspended in 1/5 of original volume 8% SDS and boiled for 30 min with the addition of water to maintain a constant volume. After cooling to room temperature, insoluble cell wall fragments were recovered by centrifugation at 3000× *g* for 15 min. The SDS treatment was repeated four times on the insoluble material to remove proteins and nucleic acids. 

Lyophilized LYSSTAPH-S was dissolved in water to obtain a concentration of 1 mg/mL. A total of 500 μL of LYSSTAPH-S solution was mixed with 2.5–3.5 mg lyophilized peptidoglycan. Enzymatic cleavage occurred for 18 h at 37 °C with agitation (600 rpm). The suspension was separated by centrifugation and the supernatant was analyzed by LC-MS technique without further treatment. 

### 4.9. Peptidoglycan HPLC-MS Analysis

HPLC-MS experiments were focused on the separation and identification of individual products of enzymatic digestion of the peptidoglycan. Chromatographic analyses were carried out on an Agilent 1100 series instrument (Agilent Technologies, Santa Clara CA, USA) coupled with a diode-array detector (Agilent Technologies, Santa Clara CA, USA) and an ion-trap mass spectrometer amaZon SL equipped with an ESI ion source (Bruker Daltonics, Fremont, CA, USA). The chromatographic separation was performed on Poroshell 120 SB-AQ column (150 × 2.1 mm; 2.7 μm) (Agilent Technologies, Santa Clara CA, USA) at 35 °C. The mobile phase consisted of eluent A: 0.1% formic acid in water and eluent B: 0.1% formic acid in methanol at a flow rate of 0.2 mL min^−1^. The gradient started at 1% of B, increased at a constant rate until 70% of B in 20 min, then held constant until 25 min, and then back to the initial 1%, and held constant until 40 min. The injected volume was 2.5 µL. The MS conditions were as follows: spray voltage, −5.0 kV; the pressure of nebulizer gas, 25 psi; the flow of dry gas, 10 L/min; the temperature of dry gas, 300 °C. The measurements were performed in Ultrascan mode in the range of m/z 70–2200. Structural analyses were based on MS data obtained in negative ion mode.

## 5. Conclusions

LYSSTAPH-S and LYSDERM-S were modified in order to improve their antimicrobial properties and stability and these antimicrobial enzymes proved to be efficient in killing of the diverse strains of methicillin-resistant *Staphylococcus aureus*. Further analyses proved no difference in the bacterial cell wall composition among the tested strains. Nevertheless, there was a significant difference in the biofilm formation among the strains. Unexpectedly, stronger biofilm producers were more susceptible to enzyme impact than the weak ones.

## Supplementary Materials

The following are available online at https://www.mdpi.com/2079-6382/9/8/519/s1, Figure S1: SDS-PAGE electrophoresis of recombinant proteins LYSDERM-S and LYSSTAPH-S. L (cell lysate), FT (flow-through), W (washed), P (pure protein), S (standard; ThermoFisher #26623). Figure S2: Antibacterial susceptibility to LYSSTAPH-S of *S. aureus* strains (sequence types ST1, ST5, ST7, ST8, ST15, ST22, ST30, ST45, ST80, ST88, ST121, ST154, ST225, ST239, ST395, and ST398) as determined by the plate lysis assay. Concentrations used: 800, 400, 200, 100, 50, 25 μg/mL. Figure S3: Antibacterial susceptibility to LYSSTAPH-S of *S. aureus* strains (sequence types 1, 5, 8, 22, 30, 45, 225, 239, 398) as determined by the plate lysis assay. Concentrations used: 800, 400, 200, 100, 50, 25 μg/mL. Figure S4: Growth curves of *S. aureus* strains (sequence types ST7, ST8, ST15, ST45, ST80, ST88, ST121, ST154, ST225, ST395, and ST398) in the presence of LYSSTAPH-S as determined by the growth kinetic assay. Concentrations ranging from 0.625 to 40 μg/mL. Figure S5: Growth curves of *S. aureus* strains (sequence types ST8, ST22, ST30, and ST225) in presence of or LYSDERM-S as determined by the growth kinetic assay. Concentrations ranging from 7.8125 to 500 μg/mL

## Patents

Utility model No. 28939/2015 and utility model No. 33404/2019.
