# Supplementary figures and images for: Enzybiotics LYSSTAPH-S and LYSDERM-S as Potential Therapeutic Agents for Chronic MRSA Wound Infections
Source: Antibiotics (Basel). 2020 Aug 15;9(8):519. doi: 10.3390/antibiotics9080519 (PMC7459665; doi:10.3390/antibiotics9080519)

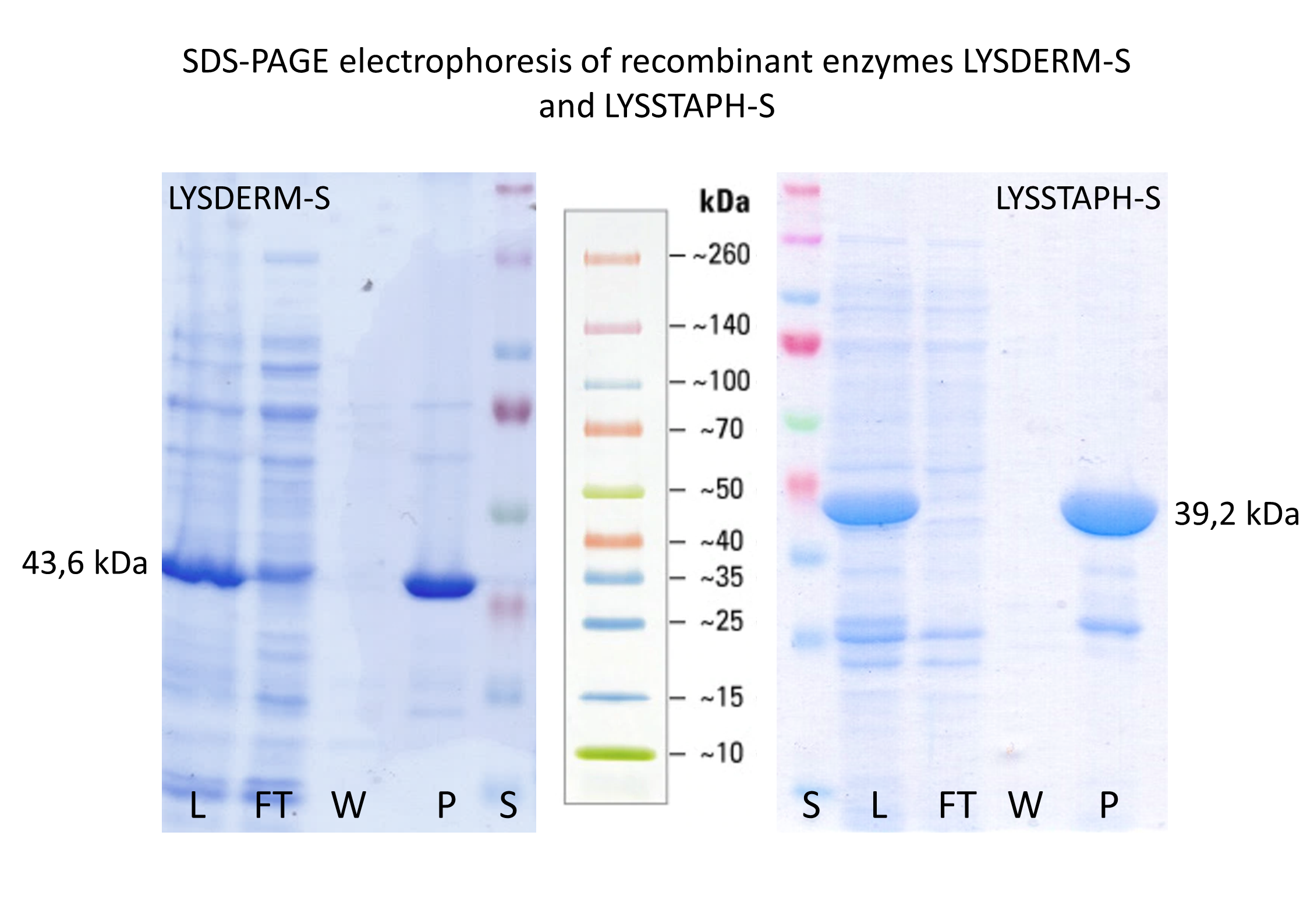

Supplement: Supplementary file 1 [file antibiotics-09-00519-s001.zip › SupplFig1.TIF]

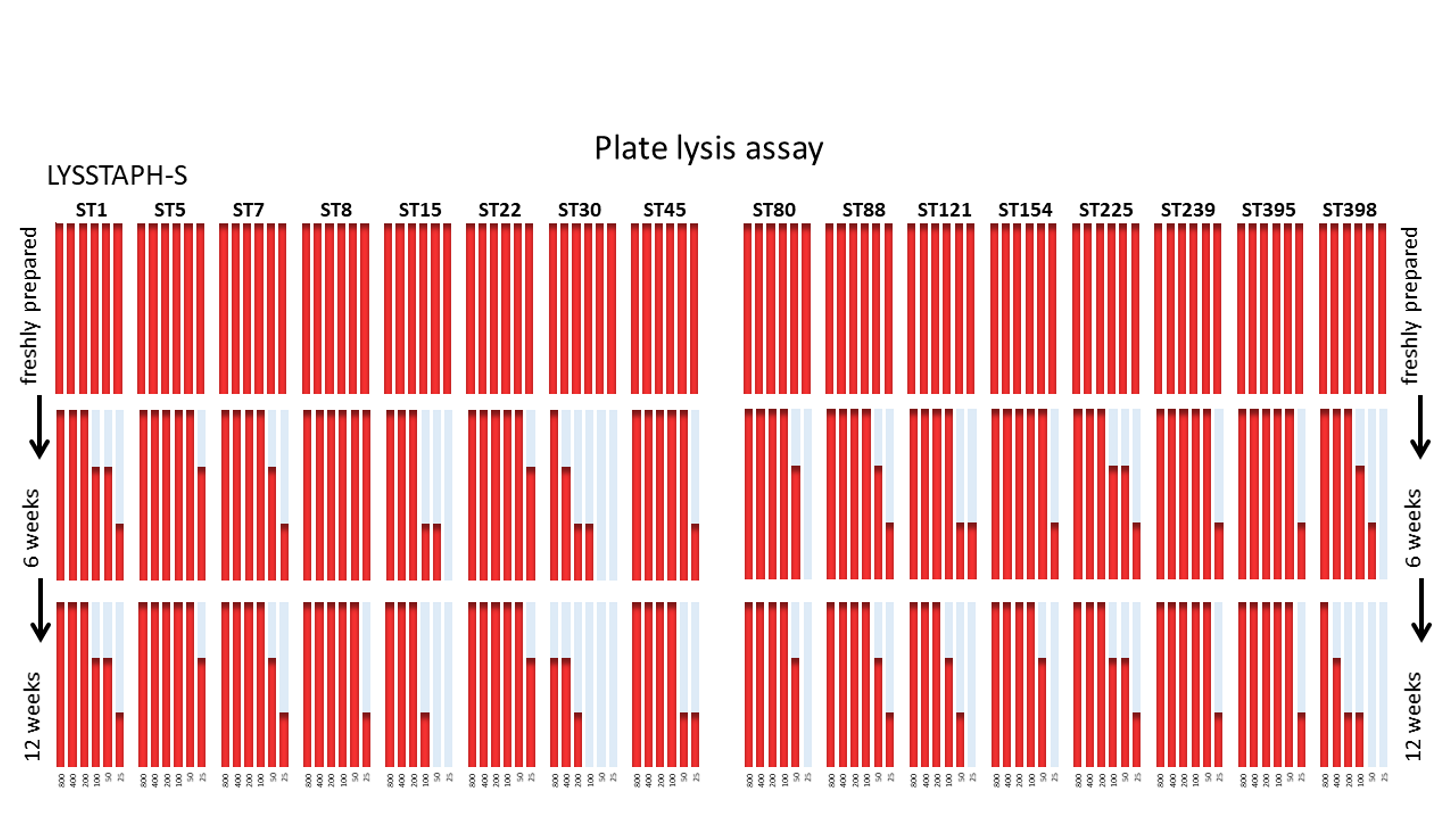

Supplement: Supplementary file 1 [file antibiotics-09-00519-s001.zip › SupplFig2.TIF]

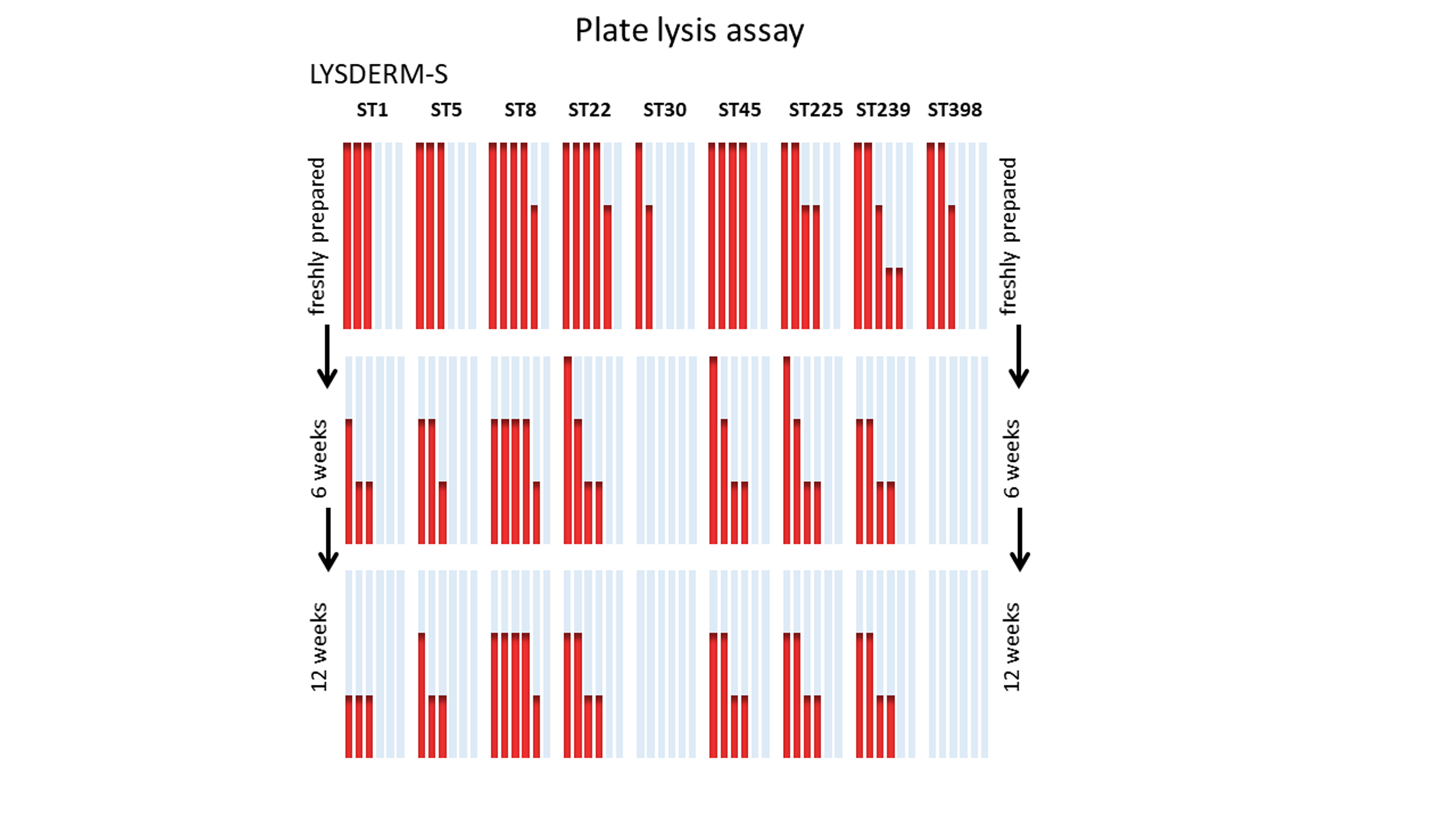

Supplement: Supplementary file 1 [file antibiotics-09-00519-s001.zip › SupplFig3.TIF]

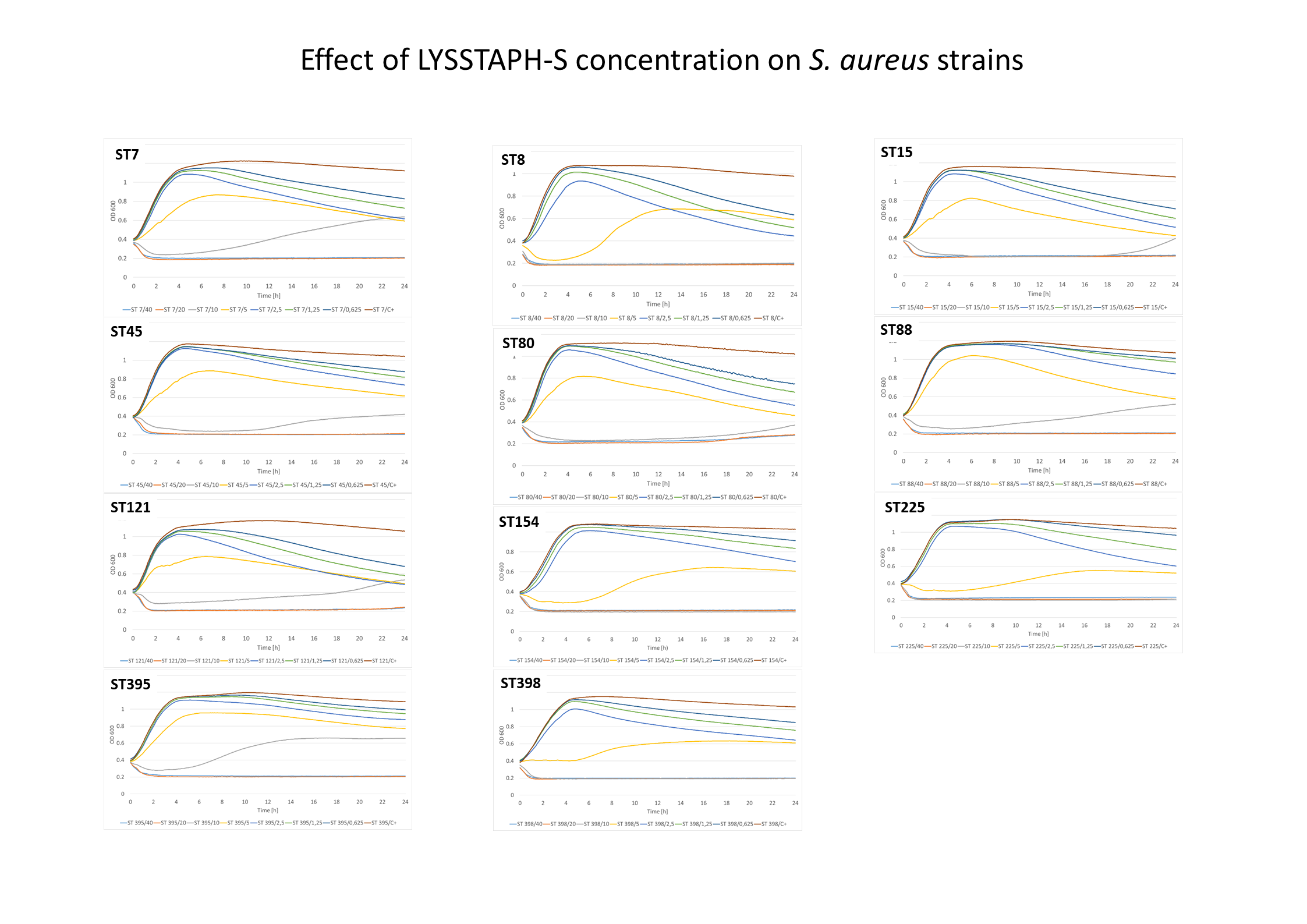

Supplement: Supplementary file 1 [file antibiotics-09-00519-s001.zip › SupplFig4.TIF]

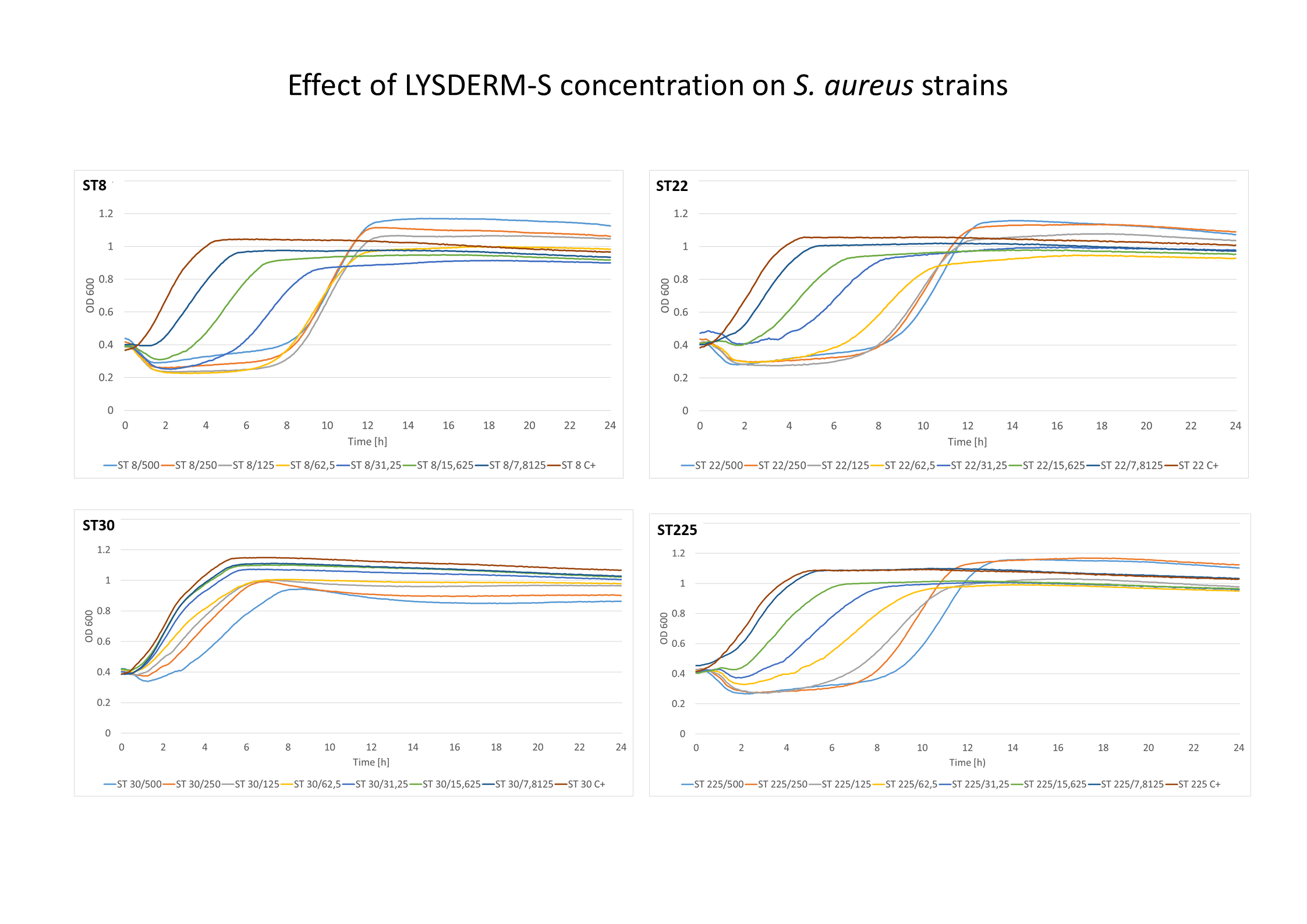

Supplement: Supplementary file 1 [file antibiotics-09-00519-s001.zip › SupplFig5.TIF]
